# National and State-Specific Attitudes toward Smoke-Free Parks among U.S. Adults

**DOI:** 10.3390/ijerph13090864

**Published:** 2016-08-31

**Authors:** Judy Kruger, Amal Jama, Michelle Kegler, Kristy Marynak, Brian King

**Affiliations:** 1 Office on Smoking and Health, National Center for Chronic Disease Prevention and Health Promotion, Centers for Disease Control and Prevention, Atlanta, GA 30306, USA; vhy4@cdc.gov (K.M.); iyn3@cdc.gov (B.K.); 2 DB Consulting Group, Atlanta, GA 30329, USA; vuj3@cdc.gov; 3 Emory University, Rollins School of Public Health, Atlanta, GA 30322, USA; mkegler@emory.edu

**Keywords:** parks, smoke-free, secondhand smoke, state, national, attitudes

## Abstract

Outdoor places, such as parks, remain a source of secondhand smoke (SHS) exposure. We assessed attitudes toward smoke-free parks among U.S. adults. Data came from the 2009–2010 National Adult Tobacco Survey, a landline and cellular telephone survey of noninstitutionalized adults aged ≥18 in the 50 U.S. states and D.C. Descriptive statistics and logistic regression were used to assess the prevalence and sociodemographic correlates of attitudes toward smoke-free parks, overall and by current tobacco use. Overall, 38.5% of adults reported favorable attitudes toward complete smoke-free parks; prevalence ranged from 29.2% in Kentucky to 48.2% in Maine. Prevalence of favorable attitudes toward smoke-free parks was higher among nonusers of tobacco (44.6%) and noncombustible-only users (30.0%) than any combustible users (21.3%). The adjusted odds of having a favorable attitude were higher among: women; Hispanics and Black non-Hispanics, American Indian and Alaska Native non-Hispanics, and other non-Hispanics; those with an unspecified sexual orientation; and those with children aged ≤17 in the household, relative to each characteristics respective referent group. Odds were lower among: any combustible tobacco and noncombustible-only tobacco users; adults aged 45–64; and those with some college or an undergraduate degree. Opportunities exist to educate the public about the benefits of smoke-free outdoor environments.

## 1. Introduction

Exposure to secondhand smoke (SHS) has multiple significant negative effects on health across the lifespan, and the U.S. Surgeon General has concluded that there is no risk-free level of SHS exposure [1,2]. Each year in the U.S., SHS exposure causes more than 41,000 deaths among nonsmoking adults and 400 deaths among infants, and approximately $5.6 billion in lost productivity [3]. Although population exposure to SHS has declined over the past two decades, many nonsmokers remain exposed to SHS in workplaces, homes, vehicles, and outdoor areas. SHS exposure in the U.S. population aged ≥3 years declined from 52.5% during 1999–2000 to 25.3% during 2011–2012; however, during 2011–2012, 58 million people were still exposed to SHS, including two in five children aged 3 to 11 years [4].

The World Health Organization Framework Convention on Tobacco Control recommends completely smoke-free environments to protect the public from SHS exposure in indoor worksites, public transport, indoor public places, and other indoor public places [5]. However, although there has been an increase in the adoption of smoke-free policies covering indoor public places, these policies do not protect people from SHS exposure in all public areas, including outdoor areas such as parks [6,7,8,9]. The implementation of policies prohibiting smoking in outdoor public places in the U.S., including parks, has increased in recent years [10,11,12,13,14,15]. As of October 2015, over 1100 municipalities in 46 states and the District of Columbia had enacted smoke-free park laws [16]. For example, New York City continues to generate success in denormalizing smoking in public places through the implementation of smoke-free laws, which has resulted in reduced smoking in parks and on beaches [17,18,19]. In addition, at the state and territory level, Oklahoma prohibits smoking on all indoor and outdoor state-owned land, Hawaii prohibits smoking in state parks, and Puerto Rico prohibits smoking in all parks [16].

Parks can be especially important places to adopt smoke-free policies because these setting are frequented by children, who are particularly susceptible to the adverse effects of SHS exposure, and who could view tobacco use as a socially acceptable behavior if exposed to it in public settings [1,2,20]. Evidence suggests that even low levels of exposure to SHS, such as that which could occur in outdoor settings, can have adverse health effects [2,21,22,23,24]. For example, a recent review examining the impact of SHS on outdoor air quality found that particulate matter with a diameter ≤2.5 μm (PM_2.5_), which is a commonly used environmental marker for SHS, can accumulate in the lungs and is associated with numerous chronic respiratory and cardiovascular health complications [25]. Smoke-free policies could help reduce SHS exposure in parks and could lead to other positive outcomes, such as increased park attendance and revenue, increased park safety, and reduced environmental hazards [26,27,28]. Smoke-free parks could also help make tobacco use less socially acceptable [26,27,28,29], reduce fire hazards, reduce health risks to children and animals from ingestion of cigarette butts [14,30], and reduce pollution generated by discarded cigarette waste [31].

Although studies have assessed attitudes toward smoke-free policies in indoor areas and outdoor areas more generally [3], few studies have assessed attitudes toward smoke-free parks [17,18,19], and none have done so using national and state representative samples of U.S. adults. To address this gap, we assessed the national and state-specific prevalence of favorable attitudes toward smoke-free parks among U.S. adults, as well as sociodemographic correlates of favorable attitudes toward smoke-free parks overall and by current tobacco use at the national level.

## 2. Materials and Methods

### 2.1. Data Source

We used data from the 2009–2010 National Adult Tobacco Survey (NATS), a national landline and cellular telephone survey of noninstitutionalized civilian adults aged 18 years or older residing in the 50 U.S. states and the District of Columbia [32]. In brief, the 2009–2010 NATS used a stratified, multistage probability design to yield data representative at both the national and state levels. A complete description of the 2009–2010 NATS methodology is available elsewhere [33]. The NATS target population was non-institutionalized adults age 18 and older and respondents were selected from two sampling frames, one for landlines and one for cell phones. Between 20 October 2009 and 28 February 2010, a total of 118,581 interviews were conducted (landline = 110,634 and cellular = 7947). The overall response rate was 37.6% (landline = 40.4% and cellular = 24.9%) [34]. State-specific response rates ranged from 28.2% in New Jersey to 49.3% in Vermont.

### 2.2. Measures

#### 2.2.1. Attitudes toward Smoke-Free Parks

Respondents were asked about their attitudes toward SF parks using the question, “Should smoking at parks...‘always be allowed’, ‘be allowed only at some times or in some places’, or ‘never be allowed’”. Respondents who answered “never be allowed” were categorized as having a favorable attitude toward completely smoke-free parks.

#### 2.2.2. Tobacco Use

Current tobacco use status was determined by respondents’ answers to questions on the current use of six tobacco products: cigarettes, cigars/cigarillos/little cigars, water pipes, pipes, chew/snuff/dip, and snus. Respondents were classified into three categories: (1) any combustible tobacco use; (2) noncombustible-only tobacco use; and (3) no tobacco use. “Any combustible tobacco use” was defined as a respondent who reported smoking at least 100 cigarettes during their lifetime and now smoked every day or some days and/or used cigars/cigarillos/little cigars, water pipes, or pipes on ≥1 day during the past 30 days. “Noncombustible-only tobacco use” was defined as using chewing tobacco/snuff/dip or snus on ≥1 day during the past 30 days, but did not use cigars/cigarillos/little cigars, water pipes, or pipes on ≥1 day during the past 30 days, and reported currently using cigarettes “not at all”. “No tobacco use” was defined as a respondent who did not ever use combustible (cigarettes, cigars/cigarillos/little cigars, water pipes, or pipes) or noncombustible (chewing tobacco/snuff/dip or snus) tobacco. It was not possible to further stratify the “no tobacco use” category by former and never use because of the threshold used to assess lifetime use, which was ≥1 for most non-cigarette products. Additionally, e-cigarette use was not assessed in the 2009–2010 NATS, and thus, these products were not included in the definitions of tobacco use; however, the extent of use of these products was limited at the time of this survey [35], so the exclusion would not be expected to meaningfully impact this measure.

#### 2.2.3. Comprehensive Smoke-Free Law Coverage

Comprehensive smoke-free law coverage was determined using the American Nonsmokers’ Rights Foundation (ANRF) U.S. Tobacco Control Laws Database, which tracks U.S. municipal, county, and state laws relating to tobacco [36]. A comprehensive smoke-free law was defined as an ordinance or regulation that prohibits smoking in all indoor areas of non-hospitality workplaces, restaurants, and freestanding bars, including attached bars or separately ventilated rooms with no exemptions based on the number of employees. Laws included in this database are identified through systematic scanning of tobacco control publications, websites and e-mail discussion lists, biannual solicitation of information from tobacco control professionals, and partnerships. Identified tobacco control laws are coded by ANRF using standardized guidelines, and population protection estimates are calculated using data from the U.S. Census. For the purposes of this study, respondents were categorized according to the proportion of state residents covered by a state and/or local comprehensive smoke-free law as of 1 July 2010 [36]. Based on the distribution of the comprehensive smoke-free law coverage, the population fell into four categories: 100% (statewide comprehensive law); 20%–99%; 1%–19%; 0% (no local or statewide comprehensive laws) [37]. These categories were selected based on the population distribution of coverage across states to ensure that statistically reliable comparisons could be made across categories.

#### 2.2.4. Sociodemographic Characteristics

Assessed sociodemographic characteristics included: sex (male or female), age (18–24, 25–44, 45–64, or ≥65 years), race/ethnicity (Hispanic and non-Hispanic white, black, Asian, American Indian/Alaska Native, Native Hawaiian/Pacific Islander, multi-race, or other); educational attainment (0–11 years (no diploma), Graduate Equivalency Degree (GED), high school graduate, some college (no degree), associate degree, undergraduate degree, or graduate degree), marital status (married/living with a partner, single/separated/divorced, or widowed), annual household income (<$20,000, $20,000 to $49,999, $50,000 to $99,999, ≥$100,000, or unspecified), U.S. Census region (Northeast, Midwest, South, or West), sexual orientation (heterosexual/straight, lesbian/gay/bisexual/transgender (LGBT), or unspecified); and if there were children aged ≤17 years living in the household (yes or no). The aforementioned characteristics were chosen because they have previously been shown to influence the prevalence of tobacco use and attitudes toward tobacco control policies [38,39].

### 2.3. Data Analysis

Data were analyzed using SAS–Callable SUDAAN 10 (RTI International, Research Triangle Park, NC, USA) and weighted to adjust for the differential probabilities of selection and response. In order to create a data set with demographics matching the U.S. population, survey weights were generated based on sex, age, race/ethnicity, marital status, and educational attainment and telephone type, by state [33]. Because the distribution of cellular telephone respondents was less than the number of landline respondents in states, to prevent large variances and ensure adequate precision for state estimates, the national and state estimates were calculated differently. For national estimates, we included both cellular telephone and landline respondents. For state-specific estimates, we included cellular telephone respondents in states (California, Florida, Georgia, Illinois, Louisiana, New Jersey, New York, North Carolina, Ohio, Oklahoma, Pennsylvania, and Texas) that had a cellular telephone sample of 200 or more respondents [33].

Descriptive analyses, including point estimates and 95% confidence intervals (CIs), were calculated overall and by current tobacco use status. In addition, multivariate logistic regression models were constructed with a favorable attitude toward completely smoke-free parks as the dependent variable. Covariates were sex, age group, race/ethnicity, educational attainment, marital status, annual household income, U.S. region, sexual orientation, whether children aged ≤17 years were living in the household, and current tobacco use status. Regression models were constructed overall, as well as by each category of tobacco use (i.e., any combustible tobacco product, noncombustible-only tobacco use, no tobacco use), each of which adjusted for the same covariates as the overall model (excluding tobacco use).

## 3. Results

### 3.1. National Prevalence of Attitudes toward Smoke-Free Parks

Nationally, 18.9% of U.S. adults reported that smoking should “always be allowed at parks”, 42.6% reported smoking should be “allowed only at some times or in some places”, and 38.5% reported smoking should “never be allowed” (Table 1). The prevalence of reporting that smoking should “never be allowed” at parks was higher among women (43.4%) than men (33.6%); among respondents with an unspecified sexual orientation (59.5%) than heterosexual/straight (38.3%) and LGBT respondents (36.2%); and among respondents with children aged ≤17 years living in the household (43.2%) than those without children living in the household (35.3%). The prevalence of reporting that smoking should “never be allowed” was lower among adults aged 45–64 (34.8%) than other age groups (18–24 = 39.7%; 25–44 = 40.8%; ≥65 = 40.0%) and residents of states in the Midwest (36.2%) than those in other regions (West = 39.6%; Northeast = 39.2%; South = 39.0%). By tobacco use status, prevalence of reporting favorable attitudes toward smoke-free parks was higher among “no tobacco users” (44.6%) and “noncombustible-only tobacco users” (30.0%) than “any combustible tobacco users” (21.3%). No difference was observed in the prevalence of reporting that smoking should “never be allowed” between those living in a state covered by a statewide comprehensive smoke-free law (37.9%) and those living in a state not covered by a comprehensive state and/or local comprehensive smoke-free law (37.6%).

### 3.2. State Prevalence of Favorable Attitudes toward Smoke-Free Parks

Prevalence of favorable attitudes toward completely smoke-free parks ranged from 29.2% in Kentucky to 48.2% in Maine (Table 2). Among “any combustible tobacco users”, prevalence of reporting a favorable attitude ranged from 11.6% in Wyoming to 33.3% in Maryland. Among “noncombustible-only tobacco users”, prevalence of reporting a favorable attitude ranged from 18.1% in Georgia to 46.2% in Mississippi. Among “no tobacco users”, prevalence of reporting a favorable attitude towards smoke-free parks ranged from 34.9% in Colorado to 53.0% in Maine.

### 3.3. Correlates of Favorable Attitudes toward Smoke-Free Parks

Table 3 shows the adjusted odds of favorability toward completely smoke-free parks, by sociodemographic characteristics and is relative to each characteristics respective referent group. Among all respondents, the odds of favorability were higher among: women (OR (odds ratio) = 1.4); black non-Hispanics (OR = 1.4); American Indian/Alaska Native non-Hispanics (OR = 1.3); other non-Hispanics (OR = 1.6); Hispanics (OR = 1.5); respondents who did not specify their sexual orientation (OR = 1.7); and adults living in households with children aged ≤17 years (OR = 1.3). The odds of favorability toward smoke-free parks were lower among: respondents aged 45–64 years (OR = 0.8); and those with some college education (OR = 0.8) or with an undergraduate degree (OR = 0.8).

Among “any combustible tobacco users”, odds of favorability towards smoke-free parks were higher among: women (OR = 1.3); black non-Hispanics (OR = 1.5); Hispanics (OR = 1.6); and those with children ≤17 living in the household (OR = 1.5); and lower among those aged 45–64 years (OR = 0.6) (Table 3). Among “noncombustible-only tobacco users”, odds were lower among Hispanics (OR = 0.1). Among “no tobacco users”, odds were higher among: women (OR = 1.4); black non-Hispanics (OR = 1.4); Hispanics (OR = 1.5); respondents who were married/living with a partner (OR = 1.2); those who did not specify their sexual orientation (OR = 1.7); and adults with children aged ≤17 years living in the household (OR = 1.2). Among “no tobacco users”, odds were lower among: those aged 45–64 years (OR = 0.8); non-Hispanics of multiple races (OR = 0.7); and respondents with some college (OR = 0.8) or an undergraduate degree (OR = 0.8).

No significant association was observed between favorability toward smoke-free parks and population level coverage of state and/or local smoke-free laws in indoor public areas, either overall or by current tobacco use status (Table 3).

## 4. Discussion

This study found that about two in five U.S. adults had a favorable attitude toward smoke-free parks during 2009–2010. However, marked variations in favorable attitudes toward smoking restrictions in parks were observed across sociodemographic groups, current tobacco use status, and states. Although awareness of the harms of SHS exposure and the benefits of smoke-free public places have increased over time [39,40], these findings suggest that there are opportunities to further raise public awareness about the public health importance and benefits of smoke-free parks. The benefits of adopting smoke-free policies in outdoor areas such as parks extend beyond the protection of children and nonsmokers from SHS exposure and can also include reductions in litter, fire risks, and the social acceptability of tobacco use [14,30,31]. A better understanding of the general public’s attitudes toward smoke-free parks may inform strategies to adopt and enforce such policies in this setting [21,26].

Irrespective of current tobacco use status, women, Hispanics, non-Hispanic racial/ethnic minorities, and adults with children aged ≤17 years in the household were more likely to favor smoke-free parks than were men, white non-Hispanics, and adults without children aged ≤17 years in the household, which is consistent with other studies [17,27]. Additionally consistent with other studies of cigarette smokers’ attitudes toward indoor smoke-free policies, as well as surveys of public favorability toward tobacco-related policies [8,38], “any combustible tobacco users” had lower levels of favorability toward smoke-free park policies than “noncombustible-only tobacco users” or “no tobacco users”. However, given that only 45% of nonusers of tobacco reported favorability toward completely smoke-free policies in parks, efforts to promote awareness of the benefits of smoke-free policies in this setting are still warranted.

No variation was observed in the odds of favorability toward smoke-free policies by the proportion of the population covered by state and/or local comprehensive smoke-free laws in indoor public areas. This finding may be the result of broader adoption of smoke-free park policies in jurisdictions across the country, irrespective of whether the jurisdiction has a comprehensive state and/or local smoke-free law. Smoke-free park policies are rapidly growing in popularity; 1101 counties contained at least one jurisdiction with a smoke-free park policy in 2015, compared to 432 counties in 2010 (ANRF database unpublished; http://no-smoke.org) [16]. Favorable attitudes towards smoke-free policies have been found to increase after the implementation of smoke-free policies, while concern about the harms of secondhand smoke have been found to decrease [39]. Therefore, support for such policies is expected to continue to increase in the future, as smoke-free policies in indoor and outdoor public places, as well as attitudes regarding the social unacceptability of smoking, continue to proliferate [38,40,41,42,43]. However, given the greater population-level protection afforded by smoke-free policies in worksites and public places, smoke-free park policies are best suited for consideration following the implementation of comprehensive smoke-free policies in all indoor public places and worksites, including restaurants and bars.

Although most respondents reported attitudes supporting some level of smoking restrictions in parks, 43% of respondents reported that smoking should be “allowed only at some times or in some places”. Future qualitative research is warranted to explore attitudes toward these partial restrictions in depth. Specific factors to explore could include whether attitudes vary depending on the park feature (e.g., playground, beach, picnic areas, trails), presence of children, or number or density of visitors. Further exploration of the factors motivating current levels of support may also be helpful, including protection of children, reduced fire risk, and concerns about litter. This deeper understanding of current attitudes toward smoke-free parks, especially among those who are receptive to partial restrictions, may offer insights into how best to inform the adoption, implementation, and sustainment of smoke-free park policies.

These findings suggest that the majority of U.S. adults support partial or complete restrictions on smoking in public parks, while opportunities exist to continue to build awareness of the harms of outdoor SHS exposure. Smoke-free policies covering settings where children live, work, learn and play—including parks—can reinforce tobacco-free norms as well as protect against SHS exposure. In contrast, permitting smoking in parks may undermine youth tobacco prevention efforts, because youth are particularly vulnerable to visual cues to smoke and to social norms [1]. As more localities embrace smoke-free policies in indoor public spaces, tobacco control stakeholders are expanding the frontier of tobacco-free environments to include public parks, as well as multiunit housing, public beaches, private vehicles, and college campuses [1,6,7]. Continued implementation of smoke-free park policies, in coordination with efforts to implement and enforce smoke-free policies in public indoor areas, could further expand the number of smoke-free environments frequented by children and improve the comprehensiveness of tobacco control efforts [1,7].

Our findings are subject to some limitations. First, these data are self-reported, and therefore, subject to social desirability bias, which could lead to overestimates of favorability. Second, these data are from 2009–2010; thus, the data may actually be underestimates that don’t reflect more recent increases in favorable attitudes toward smoke-free parks. Third, the overall response rate for NATS was 37.6% and state-specific response rate ranged from 28.2% to 49.3%, which could increase non-response bias; however, data were weighted for selection and non-response, so the impact of such bias on the reported estimates is likely minimal. Fourth, consistent with previous reports and recommendations for the 2009–2010 NATS, cellular telephone responses were excluded for states that had fewer than 200 cellular telephone respondents [33,37,38]; thus, state-specific estimates may be underrepresented for these states. Fifth, the NATS questionnaire provided limited detail about the types of parks or settings in parks for which the respondent favored smoke-free policies, such as outdoor sports stadiums and grounds, outdoor facility within hospitality venues, and playgrounds; therefore, it’s not possible to ascertain whether respondents’ favorability toward smoke-free policies may have varied based on specific locations within parks. Finally, limited sample size resulted in wide confidence intervals for some states, and for certain measures (e.g., non-combustible only tobacco use), estimates could not be presented because of statistical instability. However, all presented estimates had a relative standard error less than 30%, which is a standard and generally recognized threshold used in the scientific literature to establish statistical stability of estimates from large population-based surveys [36,38,39]. Despite these limitations, the study has many strengths, including the use of a large, nationally representative sample and the ability to assess state-specific prevalence of favorable attitudes toward smoke-free parks among U.S. adults.

## 5. Conclusions

This study is the first to comprehensively assess the prevalence and correlates of favorable public attitudes toward smoke-free parks at the national level. The findings reveal that almost 40% of U.S. adults reported favorable attitudes towards completely smoke-free parks, and an additional 42% of adults reported attitudes supporting some level of smoking restrictions in parks. However, attitudes toward complete smoking restrictions in this setting vary by sociodemographic groups, current tobacco use status, and state. Efforts to educate the public about the dangers of SHS and the benefits of outdoor smoke-free environments could help reduce the extent of SHS exposure and social acceptability of tobacco use in parks.

## Figures and Tables

**Table 1:** Attitudes toward smoking at parks, by selected characteristics—United States, National Adult Tobacco Survey (NATS) 2009–2010.

Characteristics	Attitudes toward Smoking at Parks			
	Unweighted Frequency	Should Always Be Allowed	Be Allowed only at Some Times or in Some Places	Never Be Allowed
	n	% (95% CI)	% (95% CI)	% (95% CI)
Overall	97,978	18.9 (18.4, 19.4)	42.6 (41.9, 43.3)	38.5 (37.9, 39.2)
Sex				
Male	38,908	23.5 (22.7, 24.4)	42.9 (41.8, 43.9)	33.6 (32.6, 34.6)
Female	59,070	14.3 (13.8, 14.9)	42.3 (41.5, 43.1)	43.4 (42.5, 44.2)
Age (years)				
18–24	4782	18.3 (16.6, 20.0)	42.0 (39.8, 44.2)	39.7 (37.6, 41.9)
25–44	25,961	16.3 (15.4, 17.2)	42.9 (41.7, 44.1)	40.8 (39.6, 42.0)
45–64	42,100	21.8 (21.0, 22.6)	43.5 (42.5, 44.4)	34.8 (33.8, 35.7)
≥65	25,135	19.6 (18.6, 20.6)	40.4 (39.1, 41.7)	40.0 (38.7, 41.2)
Race/ethnicity				
White, non-Hispanic	81,606	20.3 (19.7, 20.8)	44.2 (43.5, 44.8)	35.5 (34.9, 36.2)
Black, non-Hispanic	7272	17.9 (16.2, 19.5)	37.9 (35.8, 40.0)	44.2 (42.1, 46.4)
Asian, non-Hispanic	1641	6.4 ( 4.6, 8.2)	50.0 (44.1, 55.9)	43.6 (38.0, 49.3)
AI/AN, non-Hispanic	1523	24.4 (19.7, 29.2)	38.6 (33.1, 44.1)	37.0 (31.5, 42.5)
NH/PI, non-Hispanic	397	23.9 (14.3, 33.4)	42.1 (31.9, 52.2)	34.1 (24.2, 44.0)
Multi-race, non-Hispanic	1231	17.3 (13.4, 21.3)	53.5 (47.5, 59.4)	29.2 (24.3, 34.1)
Other, non-Hispanic	481	20.4 (12.6, 28.2)	35.1 (25.8, 44.4)	44.5 (34.7, 54.2)
Hispanic	3827	14.1 (12.1, 16.2)	36.4 (33.5, 39.3)	49.5 (46.5, 52.4)
Education				
0–12 years (no diploma)	6700	22.0 (20.0, 24.0)	36.3 (33.7, 38.8)	41.8 (39.2, 44.3)
Graduate Equivalency Degree	1722	26.7 (22.9, 30.4)	42.2 (37.9, 46.5)	31.1 (27.0, 35.2)
High school graduate	21,162	21.2 (20.1, 22.2)	40.8 (39.5, 42.1)	38.0 (36.8, 39.3)
Some college (no degree)	15,827	19.6 (18.4, 20.8)	44.5 (43.1, 46.0)	35.9 (34.5, 37.3)
Associate degree	14,241	18.3 (17.1, 19.5)	43.7 (42.1, 45.2)	38.0 (36.5, 39.5)
Undergraduate degree	23,150	14.3 (13.5, 15.1)	47.3 (46.0, 48.5)	38.4 (37.2, 39.6)
Graduate degree	15,176	11.6 (10.7, 12.5)	45.9 (44.4, 47.3)	42.6 (41.1, 44.0)
Marital status				
Single/separated/divorced or widowed	38,628	21.7 (20.8, 22.6)	41.5 (40.5, 42.6)	36.8 (35.7, 37.8)
Married/living with a partner	59,350	16.8 (16.2, 17.5)	43.4 (42.5, 44.2)	39.8 (39.0, 40.6)
Annual household income				
<$20,000	11,068	23.6 (21.9, 25.2)	37.2 (35.2, 39.2)	39.3 (37.2, 41.3)
$20,000–$49,999	31,669	20.3 (19.4, 21.3)	41.6 (40.4, 42.8)	38.1 (36.9, 39.2)
$50,000–$99,999	33,395	17.5 (16.7, 18.3)	45.0 (43.8, 46.1)	37.5 (36.4, 38.6)
≥$100,000	18,380	14.9 (13.8, 16.0)	45.8 (44.3, 47.2)	39.3 (37.9, 40.7)
Unspecified	3466	17.9 (15.1, 20.8)	38.1 (34.7, 41.6)	43.9 (40.3, 47.6)
U.S. region ^a^				
West	21,297	16.4 (15.2, 17.7)	44.0 (42.2, 45.8)	39.6 (37.8, 41.4)
Northeast	17,702	17.3 (16.2, 18.4)	43.5 (42.1, 44.9)	39.2 (37.8, 40.6)
Midwest	20,276	22.7 (21.7, 23.7)	41.1 (40.0, 42.3)	36.2 (35.0, 37.3)
South	38,703	18.9 (18.0, 19.7)	42.2 (41.1, 43.2)	39.0 (37.9, 40.0)
Sexual orientation				
Heterosexual/straight	94,870	18.9 (18.3, 19.4)	42.8 (42.1,43.5)	38.3 (37.6, 39.0)
LGBT	2186	23.4 (19.6, 27.2)	40.5 (36.2, 44.7)	36.2 (31.7, 40.6)
Unspecified	922	10.8 (6.9, 14.8)	29.6 (22.8, 36.4)	59.5 (52.2, 66.8)
Children ≤17 living in household				
No	67,301	21.6 (20.9, 22.3)	43.1 (42.3, 43.9)	35.3 (34.5, 36.0)
Yes	30,677	15.0 (14.2, 15.8)	41.8 (40.7, 43.0)	43.2 (42.0, 44.4)
Proportion of state population covered by a comprehensive state and/or local smoke-free law ^c^				
0%	19,299	19.2 (18.2, 20.2)	43.2 (42.0, 44.4)	37.6 (36.5, 38.8)
1%–19%	14,235	16.9 (15.6, 18.2)	41.7 (39.9, 43.6)	41.4 (39.6, 43.2)
20%–99%	17,689	19.9 (18.6, 21.2)	42.8 (41.2, 44.5)	37.3 (35.7, 38.9)
100%	44,587	19.4 (18.7, 20.2)	42.7 (41.7, 43.6)	37.9 (37.0, 38.9)
Tobacco use ^b^				
No tobacco use	78,497	13.2 (12.6, 13.7)	42.3 (41.5, 43.0)	44.6 (43.8, 45.4)
Noncombustible-only tobacco use	1818	28.1 (24.3, 32.1)	42.0 (37.9, 46.2)	30.0 (26.1, 34.2)
Any combustible tobacco use	17,068	35.1 (33.8, 36.5)	43.6 (42.2, 45.1)	21.3 (20.1, 22.6)

Abbreviations: AI/AN, American Indian/Alaska Native; CI, confidence interval; LGBT, lesbian, gay, bisexual, or transgender; NH/PI, Native Hawaiian/Pacific Islander. ^a^ *West*: Alaska, Arizona, California, Colorado, Hawaii, Idaho, Montana, Nevada, New Mexico, Oregon, Utah, Washington, and Wyoming; *Northeast*: Connecticut, Maine, Massachusetts, New Jersey, New Hampshire, New York, Pennsylvania, Rhode Island, and Vermont; *Midwest*: Illinois, Indiana, Iowa, Kansas, Michigan, Minnesota, Missouri, Nebraska, North Dakota, Ohio, South Dakota, and Wisconsin; and *South*: Alabama, Arkansas, Delaware, District of Columbia, Florida, Georgia, Kentucky, Louisiana, Maryland, Mississippi, North Carolina, Oklahoma, South Carolina, Tennessee, Texas, Virginia and West Virginia; ^b^ Any combustible tobacco use was defined as current use of at least 100 cigarettes during their lifetime, and now smoked “every day” or “some days”, and/or used cigars/cigarillos/little cigars, water pipes, or pipes on ≥1 day during the past 30 days. Noncombustible-only tobacco use was defined as use of chewing tobacco/snuff/dip or snus on ≥1 day during the past 30 days, but did not use cigars/cigarillos/little cigars, water pipes, or pipes on ≥1 day during the past 30 days, and reported currently using cigarettes “not at all”. No tobacco use was defined as a respondent who did not currently use combustible (cigarettes, cigars/cigarillos/little cigars, water pipes, or pipes) or non-combustible (chewing tobacco/snuff/dip or snus) tobacco; ^c^ Population coverage of smoke-free laws was based on the American Nonsmokers’ Rights Foundation U.S. Tobacco Control Laws Database and represents the percent of the population covered by a state and/or local comprehensive smoke-free laws as of 1 July 2010. A comprehensive smoke-free law is defined as one that prohibits smoking in all indoor areas of non-hospitality worksites, restaurants, and bars.

**Table 2:** National and state prevalence of favorable attitudes toward completely smoke-free parks, by current tobacco use status—United States, National Adult Tobacco Survey (NATS), 2009–2010.

State	Favorable Attitude toward Completely Smoke-Free Parks ^a^ (%, 95% CI)			
	Overall (*n* = 94,629)	Any Combustible Tobacco Use ^b^ (*n* = 16,193)	Noncombustible-Only Tobacco Use ^c^ (*n* = 1745)	No Tobacco Use ^d^ (*n* = 76,691)
100% Comprehensive smoke-free Law coverage ^e^				
Arizona	40.1 (35.2, 44.9)	18.4 (7.7, 29.1)	^f^	44.6 (39.3, 49.9)
Colorado	30.5 (27.1, 34.0)	16.3 (8.6, 24.0)	^f^	34.9 (31.2, 38.5)
Delaware	39.5 (35.6, 43.3)	20.0 (12.6, 27.4)	^f^	45.7 (41.5, 49.9)
District of Columbia	33.1 (28.6, 37.5)	14.7 (7.7, 21.6)	^f^	39.1 (34.2, 44.0)
Florida ^g^	40.0 (36.9, 43.1)	21.5 (15.8, 27.3)	^f^	45.5 42.1, 49.0)
Hawaii	33.2 ( 29.5, 37.0)	12.2 (5.8, 18.7)	^f^	39.8 (35.6, 44.0)
Illinois ^g^	40.2 (36.9, 43.6)	24.9 (18.0, 31.7)	^f^	45.3 (41.6, 49.1)
Iowa	40.5 (36.9, 44.2)	17.7 (9.9, 25.4)	^f^	47.3 (43.3, 51.4)
Kansas ^h^	34.1 ( 30.5, 37.8)	18.8 (9.5, 28.1)	^f^	38.8 (34.8, 42.8)
Louisiana ^g^	37.8 (35.6, 40.0)	20.3 (16.5, 24.2)	31.4 (20.3, 42.4)	46.1 (43.5, 48.6)
Maine	48.2 (44.7, 51.8)	32.5 (24.6, 40.4)	^f^	53.0 (49.1, 56.8)
Maryland	44.4 (40.3, 48.6)	33.3 (22.8, 43.7)	^f^	47.7 (43.2, 52.2)
Massachusetts	34.4 (30.7, 38.1)	18.8 (10.8, 26.8)	^f^	39.0 (34.9, 43.0)
Michigan ^h^	33.0 (29.4, 36.7)	19.5 (11.5, 27.6)	^f^	37.6 (33.6, 41.7)
Minnesota	37.5 (33.9, 41.1)	17.4 (9.8, 25.0)	^f^	41.5 (37.6, 45.5)
Montana	38.4 (34.7, 42.0)	27.0 (18.3, 35.7)	39.8 (17.2, 62.3)	41.8 (38.0, 45.6)
Nebraska	37.0 (33.4, 40.5)	16.5 (10.1, 22.9)	^f^	43.5 (39.4, 47.6)
Nevada	29.2 (25.7, 32.7)	16.0 (9.2, 22.7)	^f^	35.4 (31.2, 39.6)
New Jersey ^g^	38.4 (36.0, 40.8)	22.5 (18.0, 27.0)	^f^	42.8 (40.0, 45.5)
New Mexico	39.0 (34.6, 43.4)	23.5 (13.8, 33.2)	^f^	45.0 (40.2, 49.9)
New York ^g^	39.7 (36.5, 42.8)	22.9 (16.4, 29.3)	^f^	44.2 (40.6, 47.8)
Ohio ^g^	34.1 (31.4, 36.8)	17.8 (13.1, 22.5)	^f^	40.2 (37.0, 43.4)
Oregon ^i^	33.2 (29.5, 36.9)	20.0 (10.7, 29.2)	^f^	36.4 (32.5, 40.3)
Rhode Island	44.8 (40.8, 48.9)	31.7 (23.2, 40.2)	^f^	50.0 (45.6, 54.4)
Utah	45.0 (41.2, 48.8)	18.3 (6.8, 29.9)	^f^	49.5 (45.6, 53.4)
Vermont	41.3 (38.0, 44.7)	22.0 (14.3, 29.7)	^f^	47.0 (43.3, 50.6)
20%–99% Comprehensive smoke-free law coverage ^e^				
Alaska	34.2 (30.7, 37.6)	17.9 (11.3, 24.5)	^f^	41.0 (37.1, 44.9)
Indiana	40.8 (37.2, 44.5)	24.7 (16.5, 32.8)	^f^	47.4 (43.4, 51.3)
Kentucky	29.2 (25.6, 32.8)	15.0 (9.7, 20.3)	^f^	37.3 (32.9, 41.7)
North Carolina ^g^	37.3 (34.0, 40.5)	24.6 (17.9, 31.2)	^f^	42.8 (39.1, 46.6)
North Dakota ^j^	42.1 (38.7, 45.5)	21.0 (13.9, 28.0)	^c^	48.6 (44.8, 52.4)
South Carolina	41.9 (39.2, 44.6)	25.5 (20.6, 30.5)	^f^	48.3 (45.2, 51.4)
Texas ^g^	39.1 (36.0, 42.2)	22.0 (16.2, 27.7)	^f^	45.2 (41.6, 48.8)
Washington	34.9 (31.4, 38.4)	16.5 (9.7, 23.4)	^f^	41.1 (37.3, 45.0)
West Virginia	36.6 (32.9, 40.2)	17.2 (10.5, 23.8)	34.8 (18.3, 51.3)	44.5 (40.2, 48.8)
1%–19% Comprehensive smoke-free law coverage ^e^				
Alabama	38.7 (35.0, 42.5)	22.1 (15.6, 28.6)	^f^	46.1 (41.7, 50.4)
Arkansas	38.6 (35.4, 41.8)	24.2 (17.7, 30.7)	29.6 (16.1, 43.0)	45.8 (42.0, 49.6)
California ^g^	44.5 (41.3, 47.7)	25.3 (18.8, 31.9)	^f^	49.5 (45.9, 53.2)
Georgia ^g^	42.4 (39.8, 45.0)	23.5 (18.0, 28.9)	18.1 (9.3, 27.0)	49.8 (46.8, 52.8)
Mississippi	42.1 (37.7, 46.5)	31.9 (21.5, 42.3)	46.2 (29.4, 63.0)	46.3 (41.4, 51.3)
Missouri	32.0 (28.6, 35.4)	13.1 (7.9, 18.4)	^f^	39.3 (35.4, 43.3)
Wisconsin ^j^	34.6 (31.0, 38.1)	16.0 (6.6, 25.5)	^f^	39.5 (35.7, 43.3)
Wyoming	33.7 (29.8, 37.6)	11.6 (5.6, 17.6)	^f^	43.7 (39.2, 48.2)
0% Comprehensive smoke-free law coverage ^e^				
Connecticut	36.2 (32.2, 40.2)	16.3 ( 8.3, 24.3)	^f^	41.1 (36.6, 45.6)
Idaho	39.9 (35.4, 44.3)	31.6 (20.3, 42.9)	^f^	42.6 (37.7, 47.5)
New Hampshire	41.1 (37.7, 44.6)	24.8 (17.0, 32.6)	^f^	46.4 (42.7, 50.1)
North Carolina ^g^	37.3 (34.0, 40.5)	24.6 (17.9, 31.2)	^f^	42.8 (39.1, 46.6)
Oklahoma ^g^	37.7 (35.6, 39.9)	21.4 (17.8, 24.9)	31.3 (19.5, 43.1)	46.4 (43.8, 49.0)
Pennsylvania ^g^	40.0 (37.6, 42.3)	22.4 (17.9, 27.0)	^f^	46.2 (43.6, 48.9)
South Dakota ^j^	42.1 (38.4, 45.8)	25.1 (16.3, 34.0)	^f^	48.5 (44.5, 52.4)
Tennessee	38.3 (34.5, 42.1)	25.5 (16.8, 34.2)	^f^	44.7 (40.4, 49.0)
Virginia	37.6 (34.0, 41.3)	16.1 (10.4, 21.8)	^f^	44.9 (40.7, 49.1)
National	38.6 (37.9, 39.2)	21.3 (20.1, 22.6)	30.0 (26.1, 34.2)	44.6 (43.8, 45.4)

Abbreviation: CI, confidence interval. ^a^ Favorable attitudes toward completely smoke-free parks was defined as a response of “never be allowed” to the question, “Should smoking at parks... always be allowed, be allowed only at some times or in some places, or never be allowed”. For display purposes we have organized states based on the following categories: 100% (statewide comprehensive law); 20%–99%; 1%–19%; 0% (no local or statewide laws); ^b^ Any combustible tobacco use was defined as current use of at least 100 cigarettes during their lifetime, and now smoked “every day” or “some days”, and/or used cigars/cigarillos/little cigars, water pipes, or pipes on ≥1 day during the past 30 days; ^c^ Noncombustible-only tobacco use was defined as use of chewing tobacco/snuff/dip or snus on ≥1 day during the past 30 days, but did not use cigars/cigarillos/little cigars, water pipes, or pipes on ≥1 day during the past 30 days, and reported currently using cigarettes “not at all”; ^d^ No tobacco use was defined as a respondent who did not currently use combustible (cigarettes, cigars/cigarillos/little cigars, water pipes, or pipes) or non-combustible (chewing tobacco/snuff/dip or snus) tobacco; ^e^ Population coverage of state and/or local smoke-free laws was based on the American Nonsmokers’ Rights Foundation U.S. Tobacco Control Laws Database and represents the percent of the population covered by a state and/or local comprehensive smoke-free laws as of 1 July 2010. A comprehensive smoke-free law is defined as one that prohibits smoking in all indoor areas of non-hospitality worksites, restaurants, and bars; ^f^ Data not presented due to relative standard error ≥30%; ^g^ Calculated among landline and cellular telephone respondents. All other estimates calculated among landline respondents only; ^h^ Some states listed had implemented a comprehensive smoke-free law on the same day or around the same time as the ANR cut-off date of 1 July 2010. Michigan CSF law became effective on 1 May 2010; and Kansas CSF law became effective on 1 July 2010; ^i^ Difference between current tobacco user and nontobacco user was significant (chi-square *p* < 0.05) for all states except Oregon; ^j^ Some states listed as not previously having a comprehensive smoke-free law now have a comprehensive smoke-free law after the ANR cut-off date of 1 July 2010.Wisconsin CSF law became effective on 5 July 2010; South Dakota CSF law became effective on 10 November 2010; and North Dakota CSF law became effective on 6 December 2010.

**Table 3:** Adjusted odds of favorable attitudes toward completely smoke-free parks, by selected characteristics—United States, National Adult Tobacco Survey (NATS), 2009–2010.

Characteristics	Favorable Attitudes toward Completely Smoke-Free Parks ^a^			
	Overall (*n* = 94,629)	Any Combustible Tobacco Use ^b^ (*n* = 16,193)	Noncombustible-Only Tobacco Use ^c^ (*n* = 1745)	No Tobacco Use ^d^ (*n* = 76,691)
	AOR (95% CI) ^e^	AOR (95% CI) ^e^	AOR (95% CI) ^e^	AOR (95% CI) ^e^
Sex				
Male	1.0	1.0	1.0	1.0
Female	1.4 (1.3, 1.5)	1.3 (1.2, 1.6)	1.5 (0.8, 2.9)	1.4 (1.3, 1.5)
Age (years)				
18–24	1.0	1.0	1.0	1.0
25–44	0.9 (0.8, 1.0)	0.9 (0.7, 1.1)	1.0 (0.5, 1.9)	0.9 (0.8, 1.1)
45–64	0.8 (0.7, 0.8)	0.6 (0.5, 0.7)	0.5 (0.3, 1.0)	0.8 (0.7, 0.9)
≥65	0.9 (0.8, 1.0)	0.9 (0.6,1.1)	0.7 (0.3, 1.5)	0.9 (0.8, 1.0)
Race/Ethnicity				
White, non-Hispanic	1.0	1.0	1.0	1.0
Black, non-Hispanic	1.4 (1.3, 1.5)	1.5 (1.2, 2.0)	2.2 (1.0, 5.1)	1.4 (1.2,1.5)
Asian, non-Hispanic	1.2 (0.9, 1.5)	0.7 (0.3, 1.3)	0.2 (0.0, 1.1)	1.2 (1.0,1.6)
AI/AN, non-Hispanic	1.3 (1.0, 1.7)	1.2 (0.8, 1.9)	1.3 (0.4, 4.0)	1.4 (1.0, 1.9)
NH/PI, non-Hispanic	0.9 (0.6, 1.4)	0.5 (0.2, 1.6)	0.1 (0.0, 1.4)	1.1 (0.7, 1.8)
Multi-race, non-Hispanic	0.8 (0.6, 1.0)	1.0 (0.6, 1.7)	0.4 (0.1, 2.1)	0.7 (0.5, 0.9)
Other, non-Hispanic	1.6 (1.1, 2.4)	1.7 (0.7, 4.0)	^f^	1.5 (1.0, 2.3)
Hispanic	1.5 (1.4, 1.7)	1.6 (1.2, 2.2)	0.1 (0.0, 0.6)	1.5 (1.3, 1.7)
Education				
0–12 years (no diploma)	1.0	1.0	1.0	1.0
Graduate Equivalency Degree	0.8 (0.6, 1.0)	0.9 (0.6, 1.3)	0.7 (0.2, 3.0)	0.8 (0.6, 1.0)
High school graduate	0.9 (0.8, 1.0)	1.0 (0.8, 1.2)	1.1 (0.6, 2.0)	0.9 (0.7, 1.0)
Some college (no degree)	0.8 (0.7, 0.9)	0.9 (0.7, 1.1)	1.0 (0.5, 2.1)	0.8 (0.6, 0.9)
Associate degree	0.9 (0.8, 1.0)	0.9 (0.7, 1.1)	0.7 (0.3, 1.5)	0.9 (0.7, 1.0)
Undergraduate degree	0.8 (0.7, 0.9)	1.0 (0.8, 1.3)	0.6 (0.3, 1.3)	0.8 (0.7, 0.9)
Graduate degree	0.9 (0.8, 1.1)	0.9 (0.7, 1.3)	0.9 (0.3, 2.3)	0.9 (0.8, 1.1)
Marital status				
Single/separated/divorced or widowed	1.0	1.0	1.0	1.0
Married/living with a partner	1.1 (1.0, 1.2)	1.0 (0.8, 1.1)	1.1 (0.7, 1.7)	1.2 (1.1, 1.3)
Annual household income				
<$20,000	1.0	1.0	1.0	1.0
$20,000–$49,999	0.9 (0.8, 1.0)	0.9 (0.7, 1.1)	0.8 (0.4, 1.6)	0.9 (0.8, 1.1)
$50,000–$99,999	0.9 (0.8, 1.0)	1.0 (0.7, 1.2)	1.1 (0.5, 2.2)	0.9 (0.8, 1.0)
≥$100,000	0.9 (0.8, 1.1)	1.1 (0.8, 1.5)	1.0 (0.4, 2.2)	0.9 (0.8, 1.0)
Unspecified	1.0 (0.9, 1.2)	0.8 (0.5, 1.2)	0.8 (0.3, 2.4)	1.1 (0.9, 1.3)
U.S. region ^g^				
West	1.0	1.0	1.0	1.0
Northeast	1.1 (1.0, 1.2)	1.1 (0.9, 1.4)	1.4 (0.7, 2.9)	1.1 (1.0, 1.2)
Midwest	1.0 (0.9,1.1)	0.9 (0.7, 1.2)	1.8 (1.0, 3.1)	1.0 (0.9, 1.1)
South	1.1 (1.0, 1.2)	1.1 (0.9, 1.3)	1.4 (0.8, 2.5)	1.1 (1.0, 1.2)
Sexual orientation				
Heterosexual/straight	1.0	1.0	1.0	1.0
LGBT	1.0 (0.8, 1.2)	0.9 (0.6, 1.4)	1.4 (0.3, 6.6)	1.1 (0.8, 1.3)
Unspecified	1.7 (1.3, 2.4)	2.2 (0.9, 5.0)	0.5 (0.1, 2.1)	1.7 (1.2, 2.4)
Children ≤17 living in household				
No	1.0	1.0	1.0	1.0
Yes	1.3 (1.2, 1.4)	1.5 (1.3, 1.8)	0.8 (0.5, 1.2)	1.2 (1.1, 1.3)
Proportion of state population covered by a comprehensive state and/or local smoke-free law ^h^				
0%	1.0	1.0	1.0	1.0
1%–19%	1.0 (0.9, 1.1)	1.0 (0.8, 1.2)	1.4 (0.8, 2.5)	1.0 (0.9, 1.1)
20%–99%	1.1 (1.0, 1.2)	1.0 (0.8, 1.3)	1.1 (0.7, 2.0)	1.1 (1.0, 1.2)
100%	1.0 (0.9, 1.1)	1.0 (0.8, 1.2)	1.4 (0.8, 2.5)	1.0 (0.9, 1.1)
Tobacco use ^i^				
No tobacco use	1.0	--	--	--
Noncombustible-only tobacco use	0.6 (0.5, 0.6)	--	--	--
Any combustible tobacco use	0.3 (0.3, 0.4)	--	--	--

Abbreviations: AOR, adjusted odds ratio; AI/AN, American Indian/Alaska Native; CI, confidence interval; LGBT, Lesbian, Gay, Bisexual, or Transgender; NH/PI, Native Hawaiian/Pacific Islander. ^a^ Favorable attitudes toward completely smoke-free parks was defined as a response of “never be allowed” to the question, “Should smoking at parks... always be allowed, be allowed only at some times or in some places, or never be allowed”; ^b^ Any combustible tobacco use was defined as current use of at least 100 cigarettes during their lifetime, and now smoked “every day” or “some days”, and/or used cigars/cigarillos/little cigars, water pipes, or pipes on ≥1 day during the past 30 days; ^c^ Noncombustible-only tobacco use was defined as use of chewing tobacco/snuff/dip or snus on ≥1 day during the past 30 days, but did not use cigars/cigarillos/little cigars, water pipes, or pipes on ≥1 day during the past 30 days, and reported currently using cigarettes “not at all”; ^d^ No tobacco use was defined as a respondent who did not currently use combustible (cigarettes, cigars/cigarillos/little cigars, water pipes, or pipes) or non-combustible (chewing tobacco/snuff/dip or snus) tobacco; ^e^ Odds ratios adjusted for all other covariates listed in the table. Statistically significant odds ratios noted in bold; ^f^ Data not presented due to relative standard error ≥30%; ^g^ *West*: Alaska, Arizona, California, Colorado, Hawaii, Idaho, Montana, Nevada, New Mexico, Oregon, Utah, Washington, and Wyoming; *Northeast*: Connecticut, Maine, Massachusetts, New Jersey, New Hampshire, New York, Pennsylvania, Rhode Island, and Vermont; *Midwest*: Illinois, Indiana, Iowa, Kansas, Michigan, Minnesota, Missouri, Nebraska, North Dakota, Ohio, South Dakota, and Wisconsin; and *South*: Alabama, Arkansas, Delaware, District of Columbia, Florida, Georgia, Kentucky, Louisiana, Maryland, Mississippi, North Carolina, Oklahoma, South Carolina, Tennessee, Texas, Virginia and West Virginia; ^h^ Proportion of the state population covered by a state and/or local comprehensive smoke-free law was based on the American Nonsmokers’ Rights Foundation U.S. Tobacco Control Laws Database, and represents the percent of the population covered by state and/or local comprehensive smoke-free laws as of 1 July 2010. A comprehensive smoke-free law is defined as one that prohibits smoking in all indoor areas of non-hospitality worksites, restaurants, and bars; ^i^ Overall favorable attitude towards completely smoke-free parks was defined as a response of yes to the question “Should smoking at parks never be allowed?”.

## Abbreviations

The following abbreviations are used in this manuscript:

NATS	National Adult Tobacco Survey
SHS	Secondhand smoke
U.S.	United States
